# Assessing LLM-generated vs. expert-created clinical anatomy MCQs: a student perception-based comparative study in medical education

**DOI:** 10.1080/10872981.2025.2554678

**Published:** 2025-08-30

**Authors:** Maram Elzayyat, Janatul Naeim Mohammad, Sami Zaqout

**Affiliations:** Department of Basic Medical Sciences, College of Medicine, QU Health, Qatar University, Doha, Qatar

**Keywords:** AI-generated content, medical education, anatomy, large language models, assessment quality

## Abstract

Large language models (LLMs) such as ChatGPT and Gemini are increasingly used to generate educational content in medical education, including multiple-choice questions (MCQs), but their effectiveness compared to expert-written questions remains underexplored, particularly in anatomy. We conducted a cross-sectional, mixed-methods study involving Year 2–4 medical students at Qatar University, where participants completed and evaluated three anonymized MCQ sets—authored by ChatGPT, Google-Gemini, and a clinical anatomist—across 17 quality criteria. Descriptive and chi-square analyses were performed, and optional feedback was reviewed thematically. Among 48 participants, most rated the three MCQ sources as equally effective, although ChatGPT was more often preferred for helping students identify and confront their knowledge gaps through challenging distractors and diagnostic insight, while expert-written questions were rated highest for deeper analytical thinking. A significant variation in preferences was observed across sources (χ² (64) = 688.79, *p* < .001). Qualitative feedback emphasized the need for better difficulty calibration and clearer distractors in some AI-generated items. Overall, LLM-generated anatomy MCQs can closely match expert-authored ones in learner-perceived value and may support deeper engagement, but expert review remains critical to ensure clarity and alignment with curricular goals. A hybrid AI-human workflow may provide a promising path for scalable, high-quality assessment design in medical education.

## Background

The rapid emergence of generative artificial intelligence (AI) and large language models (LLMs) is transforming medical education. OpenAI’s ChatGPT, introduced in late 2022, gained global attention for its ability to perform diverse language tasks [1]. In the medical domain, LLMs have demonstrated surprising proficiency – ChatGPT (GPT-3.5/4), for example, achieved moderate accuracy approaching the United States Medical Licensing Examination (USMLE) pass mark without any specialized training [1]. Domain-specific models have gone further: Google’s Med-PaLM became the first to surpass the USMLE threshold on benchmark questions [2]. These developments suggest that advanced LLMs possess substantial medical knowledge and reasoning capabilities. Educators have begun exploring their use in creating educational content, such as practice problems and exam items, to supplement traditional instruction [3]. This raises a critical question: Can AI systems like ChatGPT or Google’s Gemini generate high-quality medical assessment questions comparable to those authored by human experts?

Creating effective multiple-choice questions (MCQs) remains a resource-intensive task requiring faculty time and domain expertise [4]. Although MCQs are a staple in medical assessments, crafting well-structured questions – especially in clinically relevant areas like anatomy – demands attention to realism, relevance, and cognitive complexity. LLMs offer an attractive solution: when appropriately prompted, tools like ChatGPT and Gemini can rapidly generate plausible MCQs. One study found that ChatGPT produced 50 graduate-level MCQs in just 20 minutes – a process that took experienced educators over 3.5 hours – with comparable quality across most evaluated domains [5]. These findings suggest potential for LLMs to streamline question development, enhancing efficiency without compromising standards [3,5].

Nonetheless, the use of LLMs for assessment poses challenges. AI-generated content may lack the assured accuracy of human expertise and is prone to ‘hallucinations’ or subtle factual errors. In comparative studies, ChatGPT-produced anatomy and emergency medicine MCQs contained inaccuracies in about 6% of items, slightly higher than the 4% observed in faculty-authored questions [4]. Some items also featured misaligned or implausible distractors [4]. Furthermore, LLMs often generate questions with limited cognitive depth, tending toward Bloom’s lower-order categories (‘remember/understand’), whereas faculty-created items more frequently assess application and analysis [4]. Not all LLMs perform equally – ChatGPT (GPT-4), for instance, achieved 70% accuracy on medical exam questions, outperforming Google’s Gemini, which scored around 50% [6]. Such variation underscores the need for cautious implementation and thorough vetting of AI-generated questions. Multiple reviews emphasize that while LLMs can support question development, expert oversight remains essential to ensure validity and mitigate risks [7–9].

High-quality MCQs are defined by several well-established principles. Realistic clinical scenarios, clarity in wording, plausible distractors, interdisciplinary integration, and conceptual depth are essential characteristics [10–12]. Poorly constructed items – those with vague stems or implausible answers – can mislead students, distort exam scores, and undermine fairness [11,12]. Integration of disciplines (e.g., linking anatomical structures with clinical findings) and the targeting of higher-order thinking (per Bloom’s taxonomy) are especially valuable in modern medical education [10,13]. These criteria set a high bar that AI-generated questions must meet to be educationally sound.

While early studies such as those by Cheung et al. and Law et al. evaluated AI-generated questions in general medical contexts, more recent work has examined performance in preclinical subjects like anatomy [4,5]. For instance, Kıyak et al. piloted ChatGPT-generated anatomy questions, finding acceptable psychometric properties when trialed on students – though expert refinement was still needed [14]. Similarly, comparisons in physiology have revealed differences in distractor quality and cognitive alignment, reinforcing the importance of learner-centered evaluation [15]. A 2024 systematic review noted that few studies had directly compared AI- and faculty-generated MCQs in controlled settings, highlighting a critical gap [7,16].

This study addresses that need by focusing on clinical anatomy, a foundational discipline that presents unique demands in MCQ design – precision, spatial reasoning, and clinical contextualization. We compare AI-generated anatomy MCQs (using ChatGPT/Gemini) to expert-written items across dimensions such as realism, clarity, distractor quality, interdisciplinary integration, and cognitive depth. By evaluating both expert ratings and item performance with students, we aim to assess whether LLM-generated questions meet the pedagogical standards expected in medical education. Our findings will inform best practices for incorporating AI in assessment – identifying strengths, limitations, and how human-AI collaboration can best support high-quality question development.

## Methods

### Study design

This study adopted a cross-sectional, mixed-methods design to evaluate and compare the quality of clinical anatomy MCQs generated by large language models (LLMs) – namely ChatGPT 3.5 (OpenAI) and Gemini 1 (Google DeepMind) – against those created by several experienced academic anatomists for 6 medical modules using principles of Team-Based Learning (TBL). To put it simply, TBL in anatomy uses pre-class preparation, individual and team assessments, and clinically focused group application exercises to promote deep understanding and collaborative problem-solving [17]. In this study, the MCQs generated by ChatGPT and Gemini were developed using a standardized prompt to ensure consistency across both platforms. The prompt was carefully crafted to reflect explicit instructions aligned with United States Medical Licensing Examination and National Board of Medical Examiners (USMLE/NBME-style) expectations, asking each model to create six long-form, clinically oriented MCQs based on specified learning objectives within a given anatomical system. Each question was required to include five answer choices, a clear correct response, and detailed explanations justifying both the correct and incorrect options. Importantly, both LLMs were used in their native, unmodified forms without fine-tuning or constraints tied to curricular content. This design choice allowed us to evaluate the models’ baseline capabilities in generating exam-quality questions and their alignment with established assessment standards in medical education. The primary objective was to assess undergraduate medical students’ perceptions of each MCQ set across key pedagogical dimensions, including clarity, clinical realism, scientific accuracy, cognitive difficulty, and overall learner-perceived educational value.

The mixed-methods approach enabled both quantitative comparisons of student responses and qualitative analysis of open-ended feedback, providing a comprehensive understanding of the relative strengths and limitations of AI-generated versus expert-authored questions.

For each anatomical system, three distinct MCQ sets were generated between March 16–20, 2024:

- Set I: Generated using ChatGPT 3.5 (OpenAI)
- Set II: Authored by an academic anatomist utilizing TBL methodology
- Set III: Generated using Gemini 1 (Google DeepMind)

Each set contained 3 MCQs based on clinically relevant anatomical vignettes, designed in accordance with best practices in MCQ construction, including plausible distractors and context-rich scenarios. All sets were anonymized and randomized in order of presentation to minimize bias.

Participants selected one anatomy module (e.g., gastrointestinal) and completed its associated MCQ sets. They then filled out an evaluation form comprising 17 Likert-type items measuring clarity, difficulty, scientific accuracy, and educational utility. An optional open-text section invited narrative feedback on their experience with the different MCQ sets.

### Study population

Participants included Year 2 to Year 4 students enrolled in the Doctor of Medicine (MD) program at Qatar University College of Medicine. Eligibility required prior completion of at least one of the core clinical anatomy modules: cardiovascular, respiratory, gastrointestinal, renal, endocrine, or nervous systems.

A total of 48 complete responses were collected, with the highest participation from students enrolled in the cardiovascular module (*n* = 16). Participants were recruited through institutional email announcements and course-specific communication channels. Participation was voluntary, and all respondents provided informed electronic consent. Ethical approval was obtained from the Qatar University Institutional Review Board (QU-IRB; Approval No. 103/2025-EM), and all data were collected and stored in accordance with institutional confidentiality and data protection standards.

### Data collection and analysis

Quantitative data were exported from Google Forms into Microsoft Excel for processing. Statistical analyses were conducted using GraphPad Prism version 10.1. Descriptive statistics (frequencies and percentages) were computed to summarize the distribution of responses. To examine differences in response distributions across the three MCQ sources, a Chi-square test of independence was applied. A p-value < 0.05 was considered statistically significant.

Although open-ended feedback was optional, a small number of participants (*n* = 8) provided narrative comments. These responses were reviewed manually and analyzed descriptively to identify key themes and illustrative concerns. Given the limited volume of qualitative data, a grounded theory approach was not pursued. Instead, emphasis was placed on capturing representative insights that complemented the quantitative findings.

All figures were created using Adobe Photoshop version 25.1.1 and ChatGPT-4o for layout optimization and visualization consistency.

### Inclusion and exclusion criteria

#### Inclusion criteria

- Enrollment in Year 2, 3, or 4 of the MD program
- Completion of at least one of the following anatomy modules: cardiovascular, respiratory, gastrointestinal, renal, endocrine, or nervous system
- Ability to provide informed consent and complete the online evaluation

#### Exclusion criteria

- Students under 18 years of age
- Incomplete or duplicate responses
- Inability to access or complete the online form within the data collection period

## Results

### Participant demographics

**Figure 1.**
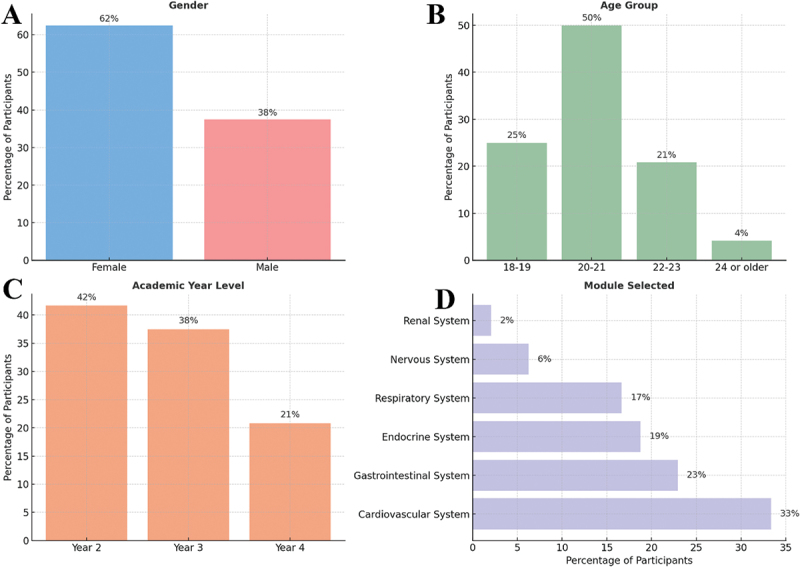
Participant demographics. the figure summarizes the distribution of participants based on gender (A), age group (B), academic year level (C), and selected clinical anatomy module (D). Percentages are calculated relative to the total number of participants (*N* = 48). Data highlights a diverse sample of undergraduate medical students contributing to the comparative evaluation of MCQs.

A total of participants from various academic levels and modules contributed to the evaluation (Figure 1). The majority were female (62%) compared to 38% male (Figure 1(A)). Most students were aged 20–21 years (50%), followed by 18–19 years (25%) and 22–23 years (21%), while only 4% were 24 years or older (Figure 1(B)). The cohort included 20 students across Year 2 (42%), 18 students across Year 3 (38%), and 10 students across Year 4 (21%) academic levels (Figure 1(C)). In terms of module selection, the highest participation was observed from students enrolled in the Cardiovascular System (33%), followed by the Gastrointestinal (23%) and Endocrine (19%) systems (Figure 1(D)).

### Comparative evaluation of MCQ sets

**Figure 2.**
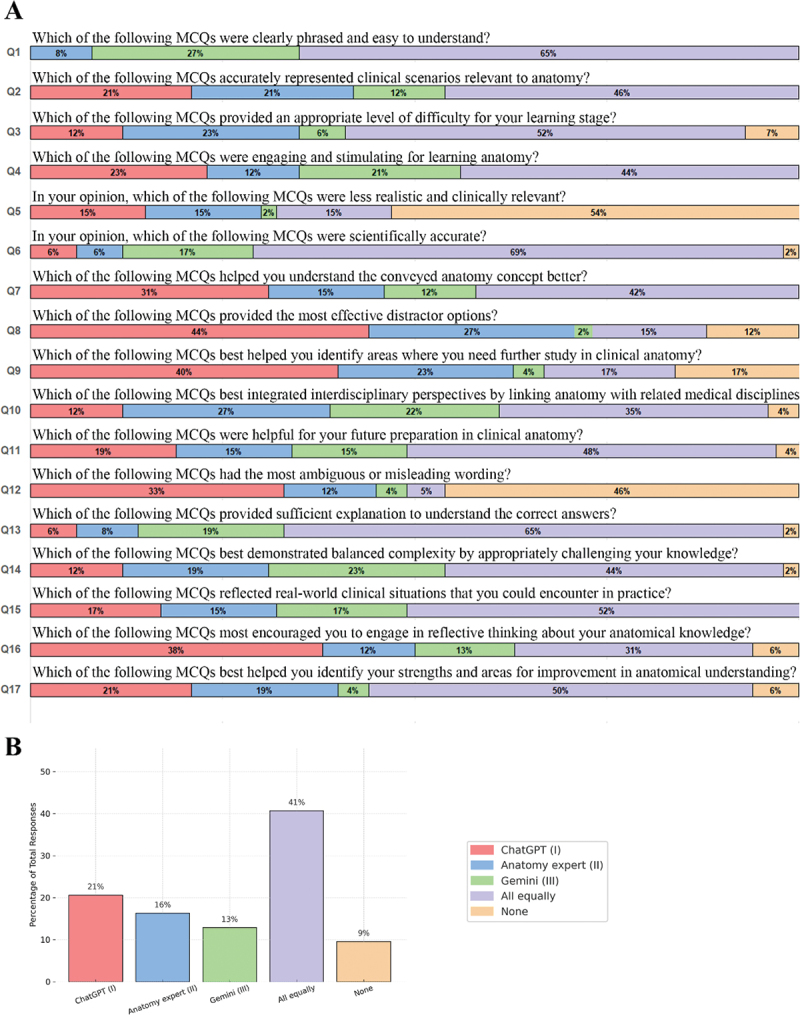
Learner-perceived quality of LLM- and expert-generated clinical anatomy MCQs. (A) Each horizontal bar represents a specific quality dimension (e.g., clarity, realism, difficulty) used to assess MCQs generated by ChatGPT (I), Gemini (III), or a human anatomy expert (II). Students selected one preferred MCQ per criterion, or chose ‘all equally’ or ‘none’ when applicable. The percentages reflect aggregated selections from all participants. Full criterion descriptions are displayed directly next to each bar. (B) Summary bar chart presenting the overall distribution of all responses aggregated across the 17 criteria, highlighting the relative preference for each source. Percentages reflect the proportion of total selections (*N* = 816 total responses).

The comparative evaluation of the three MCQ sources – ChatGPT (I), Anatomy Expert (II), and Gemini (III) – revealed clear differences in student preferences (Figure 2(A)). Across the 17 evaluation criteria, the most common response was ‘All equally’ (40.7%), indicating that students often perceived the MCQs from all three sources as comparably effective. Among the individual sources, ChatGPT received the highest proportion of preferred responses (20.6%), followed by the Anatomy Expert (16.3%) and Gemini (12.9%). The ‘None’ option was selected in 9.6% of responses (Figure 2(B)).

Item-level analysis showed that ‘All equally’ was the dominant choice for criteria such as Q1 (clarity of phrasing), Q3 (appropriate difficulty), Q6 (scientific accuracy), and Q13 (sufficiency of explanation), suggesting strong performance by all sources on foundational pedagogical standards. In contrast, ChatGPT received relatively higher preference in criteria related to critical thinking and engagement, particularly Q8 (distractor quality) and Q9 (identifying knowledge gaps). Gemini and the Anatomy Expert each demonstrated more limited item-specific strengths, with Gemini occasionally outperforming in engagement criteria (e.g., Q4), and the expert being slightly more favored in Q2 (clinical realism) and Q14 (balanced complexity).

### System-level trends in source preference

**Figure 3.**
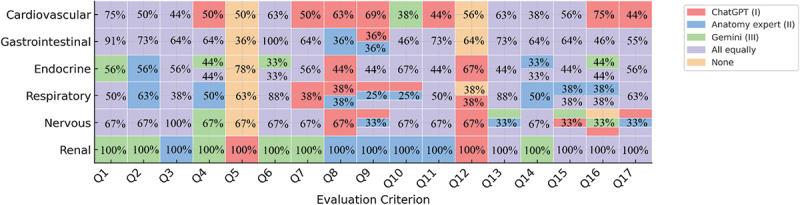
Most preferred MCQ source by system and evaluation criterion. each colored cell represents the most frequently selected MCQ origin (ChatGPT, anatomy expert, Gemini, all equally, or none) for a specific anatomical system and evaluation question (Q1–Q17). In cases of equal highest selections, the cell is split proportionally among the top sources. This heatmap reflects participants’ preferences across six anatomical systems: cardiovascular (*n* = 16), gastrointestinal (*n* = 11), endocrine (*n* = 9), respiratory (*n* = 8), nervous (*n* = 3), and renal (*n* = 1).

When stratified by clinical system, distinct patterns of MCQ source preference emerged (Figure 3). ChatGPT (I) was most frequently preferred in the Cardiovascular and Nervous systems, especially for evaluation criteria addressing distractor options and gaps in knowledge (e.g., Q8 and Q9, respectively). The Anatomy Expert (II) was more consistently selected in the Renal and Respiratory systems, particularly for promoting deeper analytical thinking by challenging understanding, identifying knowledge gaps, and integrating interdisciplinary perspectives (e.g., Q8, Q9, and Q10, respectively). Gemini (III) received more favorable ratings in the Renal and Endocrine systems, especially for clarity, engagement & scientific accuracy (e.g., Q1, Q4 and Q6, respectively).

In several cells, two or more sources shared the highest number of responses, and these were represented as vertically split cells in the heatmap to reflect equal preference. This visual representation underscored that student judgments varied not only by source but also by anatomical content and question type.

A chi-square test of independence confirmed that the distribution of student responses varied significantly across sources and criteria (χ^2^ (64) = 688.79, *p* < .001), indicating that students were able to differentiate between question sets based on specific content and educational quality markers.

### Qualitative feedback

While optional narrative responses were limited, several noteworthy themes emerged. Some students expressed concern about AI-generated MCQs being overly complex or misaligned with their learning stage, suggesting the need for faculty oversight in ensuring relevance and appropriateness. For example, one Year 2 participant noted: *‘Please if you do use AI to make questions make sure to evaluate its difficulty and relevance to your lectures.’*

Another student highlighted the challenge of questions containing multiple plausible distractors, which can lead to unnecessary confusion if not properly contextualized. Others commented positively on the role of concept mapping and interdisciplinary integration, noting improved recall and engagement. A few responses suggested that some MCQs leaned more toward pathology than anatomy, calling for more discipline-specific balance. Finally, a recurring sentiment emphasized the importance of clinical relevance in anatomy teaching, aligning with the broader goals of integrated medical education.

## Discussion

Our findings indicate that well-crafted AI-generated questions can closely approach the quality of those written by human experts. Across 17 evaluation criteria, students most frequently rated the ChatGPT, Gemini, and expert-authored anatomy questions as ‘all equally’ effective (41%), suggesting general parity in learner-perceived educational value. This aligns with prior reports showing that LLM-generated items can match faculty-written questions in overall quality [5,18]. For instance, Cheung et al. found no significant differences in quality between ChatGPT- and professor-authored MCQs, aside from a slight gap in contextual relevance [5]. Our study extends this by directly capturing student perspectives – many students perceived AI- and expert-written questions as equally clear, accurate, and appropriately challenging. Encouragingly, fewer than 10% of responses selected ‘None,’ indicating that all question sets were generally seen as acceptable.

While overall equivalence was observed, nuanced differences emerged. ChatGPT questions were often preferred for helping students identify knowledge gaps and offering challenging distractors, whereas expert-written items stood out for clinical relevance and promoting higher-order thinking. This contrast reflects complementary strengths. Notably, some students felt AI questions pushed them to reason through scenarios – suggesting that with thoughtful prompting, LLMs can engage deeper cognitive processing. This contrasts with previous concerns that AI tends to generate lower-level recall items [4,19]. Law et al., for instance, found ChatGPT’s questions mainly targeted lower-order cognition, while human-written items focused more on higher-order skills [4]. However, our findings echo emerging evidence that AI can generate complex, scenario-based items when guided appropriately. Kıyak et al. recently showed that ChatGPT could produce case-based anatomy templates with acceptable psychometric properties, supporting its potential to assess higher-level understanding when properly refined [14].

At the same time, the expert-authored questions’ strength in clarity and clinical alignment reflects the continued value of experienced educators. Human item writers are better attuned to precise wording and effective distractor design, enhancing both validity and learner comprehension [11,12,20]. This may explain why students rated expert questions as clearest and most pedagogically sound. These findings mirror those by Cheung et al. and Law et al., who noted minor flaws in AI-generated content – such as factual inaccuracies or off-topic material – that were absent in expert questions [4,5]. Similarly, Laupichler et al. found that expert-generated MCQs showed higher discriminatory power than ChatGPT’s, reinforcing the need for human oversight in final item curation [21].

Our analysis also found significant variation in student preferences across question sources (χ^2^ test *p* < 0.001). ChatGPT-generated items were particularly favored in modules like cardiovascular and nervous systems for their integrative style, while expert questions stood out in areas like renal and respiratory anatomy for their clarity and rationale. Gemini questions received mixed but generally favorable feedback, especially in renal and endocrine systems. These trends align with existing literature comparing LLM performance: GPT-4 (ChatGPT) often outperforms Gemini on medical knowledge tasks [2,6,22], and studies like Rossettini et al. support this model-specific variance [23]. It suggests that different LLMs may excel in different domains, an avenue worth exploring further [4,24].

Student feedback added important context. Some raised concerns about difficulty calibration and alignment with the curriculum, noting that AI-generated questions were sometimes too complex or tangential. This mirrors findings by Law et al., who reported a higher incidence of inappropriate difficulty in ChatGPT questions [4]. Students also noted that distractors in AI questions could sometimes be confusing or overly broad, reinforcing the importance of human validation. Well-designed MCQs require not only plausible distractors but also clarity in ensuring a single best answer, something AI doesn’t consistently deliver without oversight [11,12].

On the positive side, students appreciated that some AI-generated questions prompted interdisciplinary thinking and concept integration. Comments highlighted improved engagement when questions involved clinical reasoning or ‘concept mapping.’ These insights support the potential of LLMs to enrich assessments when guided effectively, especially for scenario-based learning [10,13]. However, a few students cautioned that some items drifted too far into pathology, underscoring the need for careful prompt design and post-editing to maintain focus.

Our results support the growing consensus that AI can be a powerful tool to augment assessment design, especially when integrated into a structured human-in-the-loop workflow. Recent work by Kıyak et al. (2025) provides a strong model for this: using AI to generate question templates, which are then refined by educators, offers a scalable way to harness AI efficiency while safeguarding quality [25]. This hybrid model – rapid AI generation followed by expert review – can reduce workload while maintaining high standards, a point emphasized by systematic reviews urging for quality control in AI-assisted content [7,8]. Our student-centered evaluation reinforces this: *AI can drastically accelerate item creation, but the final curation by knowledgeable faculty is what ensures the questions are pedagogically sound and fair*. Similar findings were reported in radiography education, where ChatGPT-generated MCQs matched faculty-authored items in difficulty but showed slightly lower discrimination indices, reinforcing the need for human oversight in ensuring assessment quality [26].

For practical implementation, educators could use LLMs to draft questions across a topic outline, then vet them using structured checklists and pilot testing. This iterative approach could yield high-quality, psychometrically sound questions over time. Providing faculty with prompt engineering guidance and frameworks for reviewing AI output will be essential to ensure consistency and safety in high-stakes environments. Institutions might also consider review workflows or trial phases for any AI-derived content [27–29].

Finally, our study has limitations. It was conducted at a single institution with 48 students in one subject area, so broader generalizability is limited. Future studies should explore this topic across specialties, learner levels, and institutions, incorporating both perception and performance data. The evolving nature of LLMs also warrants continuous evaluation of newer models and their outputs. Moreover, questions around academic integrity, ethical considerations, and student attitudes toward AI-generated assessments remain important areas for future inquiry [30,31].

In conclusion, our comparative study offers evidence that LLM-generated anatomy MCQs – when appropriately guided and reviewed – can match human-crafted questions in many respects and even excel in certain areas like promoting complex reasoning. At the same time, the distinct strengths of human expertise (in clarity, accuracy, and nuanced alignment with curriculum) remain critical to producing the highest-quality exam items. The path forward in medical education will likely not be an ‘AI vs. expert’ dichotomy, but rather a synergy: leveraging AI’s efficiency and creativity *alongside* human judgment to enhance assessment design.

## Data Availability

The data supporting the findings of this study are available from the corresponding author upon reasonable request.
